# Corrigendum: Correction to manuscripts describing the fatty acid composition of organisms submitted to IJSEM between 2014 and 2021

**DOI:** 10.1099/ijsem.0.005162

**Published:** 2022-02-10

**Authors:** The Microbiology Society

**Affiliations:** ^1^​ 14–16 Meredith Street, London, EC1R 0AB, UK

The previously published articles listed in the References [1–50] contained the phrase ‘Summed features are groups of two or three fatty acids that are treated together for the purpose of evaluation in the MIDI system and include both peaks with discrete ECLs as well as those where the ECLs are not reported separately’, which was taken from and consequently should have cited the following article:

**‘Montero-Calasanz MC, Göker M, Rohde M, Spröer C, Schumann P** ***et al.** Chryseobacterium hispalense* sp. nov., a plant-growth-promoting bacterium isolated from a rainwater pond in an olive plant nursery, and emended descriptions of *Chryseobacterium defluvii *, *Chryseobacterium indologenes *, *Chryseobacterium wanjuense* and *Chryseobacterium gregarium *. *Int J Syst Evol Microbiol* 2013; 63:4386–4395.'
